# Crystal Structure and Immunogenicity of the DS-Cav1-Stabilized Fusion Glycoprotein From Respiratory Syncytial Virus Subtype B

**DOI:** 10.20411/pai.v4i2.338

**Published:** 2019-12-11

**Authors:** M. Gordon Joyce, Amy Bao, Man Chen, Ivelin S. Georgiev, Li Ou, Tatsiana Bylund, Aliaksandr Druz, Wing-Pui Kong, Dongjun Peng, Emily J. Rundlet, Joseph G. Van Galen, Shuishu Wang, Yongping Yang, Baoshan Zhang, Gwo-Yu Chuang, Jason S. McLellan, Barney S. Graham, John R. Mascola, Peter D. Kwong

**Affiliations:** 1 Vaccine Research Center, National Institute of Allergy and Infectious Diseases, National Institutes of Health, Bethesda, Maryland; 2 Henry M. Jackson Foundation for the Advancement of Military Medicine, Inc., Bethesda, Maryland; 3 Vanderbilt Vaccine Center, Vanderbilt University Medical Center, Nashville, Tennessee; 4 Department of Pathology, Microbiology, and Immunology, Vanderbilt University Medical Center, Nashville, Tennessee; 5 Department of Electrical Engineering and Computer Science, Vanderbilt University, Nashville, Tennessee; 6 Department of Molecular Biosciences, College of Natural Sciences, The University of Texas at Austin, Austin, Texas

**Keywords:** antigenic site, crystal structure, epitope, fusion glycoprotein, immunogenicity, neutralization, RSV subtype, vaccine

## Abstract

**Background:**

Respiratory syncytial virus (RSV) subtypes, A and B, co-circulate in annual epidemics and alternate in dominance. We have shown that a subtype A RSV fusion (F) glycoprotein, stabilized in its prefusion conformation by DS-Cav1 mutations, is a promising RSV-vaccine immunogen, capable of boosting RSV-neutralizing titers in healthy adults. In both humans and vaccine-tested animals, neutralizing titers elicited by this subtype A DS-Cav1 immunogen were ~ 2- to 3-fold higher against the homologous subtype A virus than against the heterologous subtype B virus.

**Methods:**

To understand the molecular basis for this subtype difference, we introduced DS-Cav1 mutations into RSV strain B18537 F, determined the trimeric crystal structure, and carried out immunogenicity studies.

**Results:**

The B18537 DS-Cav1 F structure at 2-Å resolution afforded a precise delineation of prefusion F characteristics, including those of antigenic site Ø, a key trimer-apex site. Structural comparison with the subtype A prefusion F indicated 11% of surface residues to be different, with an alpha-carbon root-mean-square deviation (RMSD) of 1.2 Å; antigenic site Ø, however, differed in 23% of its surface residues and had an alpha-carbon RMSD of 2.2 Å. Immunization of vaccine-tested animals with DS-Cav1-stabilized B18537 F induced neutralizing responses ~100-fold higher than with postfusion B18537 F. Notably, elicited responses neutralized RSV subtypes A and B at similar levels and were directed towards both conserved equatorial and diverse apical regions.

**Conclusion:**

We propose that structural differences in apical and equatorial sites–coupled to differently focused immune responses–provide a molecular explanation for observed differences in elicited subtype A and B neutralizing responses.

## INTRODUCTION

Respiratory syncytial virus (RSV) was first identified by Chanock and coworkers in 1957 [1, 2] as the etiological agent responsible for the majority of acute respiratory infections in children. In the United States, RSV is the leading cause of pediatric intensive care hospitalizations [3]. Worldwide, RSV annually causes approximately 3 million acute lower respiratory infections in children and is typified by recurrent infections in infants [4]. Two major RSV subtypes, A and B, circulate with 1-2 epidemic periods per year depending on the climate [5, 6]. Subtype A infections appear to be more prevalent, with disease from both subtypes of similar but variable severity [7, 8]. The RSV fusion (F) glycoprotein is a dominant antigen and a prominent vaccine target [9–11]. Although most subtyping is done by sequencing of the RSV G glycoprotein, molecular phylogeny analysis of RSV F from circulating strains has helped to define RSV evolution and subtype prevalence over time [12–14]. A review by Melero and Moore [15] summarizes the differences between RSV subtype infection and the resulting effect on pathogenesis and immunity. RSV infection tends to display a regular pattern of periodicity of subtype A or B dominance in a given geographic area [16–22] with adult re-infection rates of 5%-10% [23–25]. Analysis of infection and re-infection, from a birth cohort in rural Kenya or over 8 seasons of RSV infection in Japan in a pediatric cohort, shows that 80% of RSV re-infections are due to either subtype or genotype differences [26, 27].

Analysis of human sera indicates that the majority of neutralizing antibodies elicited by natural RSV infection target the prefusion form of the F glycoprotein [28, 29], which is metastable and transitions to a more stable postfusion form. The prefusion conformation can be stabilized by the structure-based design of disulfide (DS) and cavity-filling (Cav1) mutations [30] and further stabilized with additional mutations [31, 32]. These mutations introduce covalent linkages or steric hindrance that prevent structural rearrangements required for the transition from prefusion to postfusion. A DS-Cav1-stablilized subtype A RSV F elicited levels of RSV-neutralizing antibodies in mice and macaques many times the protective threshold [30]; however, the level of elicited neutralization against a subtype B virus was only one-third the level observed against the homologous subtype A strain. In a phase I trial in adult humans [33] immunizations with a DS-Cav1-stabilized subtype A RSV F induced a higher vaccine-induced serum neutralization response against subtype A virus than subtype B virus, similar to that observed in animal models.

Several RSV-targeting monoclonal antibodies that are subtype specific, such as the highly potent monoclonal antibodies RSE20 [34] and 5C4 [35], have been identified. There are substantial differences in subtype neutralization elicited by RSV F stabilized in the prefusion conformation [35, 36], indicating that control of RSV disease may require an improved understanding of F subtype differences. To provide structural and immunogenic bases of F subtype differences, we crystallized and determined the structure of a soluble prefusion RSV F from subtype B strain B18537, stabilized by DS-Cav1 mutations. We compared this structure to prior DS-Cav1 structures [30, 37], and analyzed sequence diversity, B factor diversity, and structural variations at the apical and equatorial antigenic sites. We also assessed the immunogenicity of this subtype B prefusion-stabilized F trimer. The results define RSV F subtype differences at the atomic-level and provide insight into subtype-specific immunogenicity of the prefusion RSV F glycoprotein.

## MATERIALS AND METHODS

### Cell Lines, Media, and Antibodies

We purchased 293F human embryonic kidney cell lines from Invitrogen (Carlsbad, CA) which were maintained in Freestyle 293 expression medium (Invitrogen). Monoclonal antibodies used in binding studies, AM22, D25, 5C4, motavizumab, and MPE8, were expressed by transient co-transfection of both the heavy and light chain gene-containing plasmids into 293F cells in suspension culture at 37°C. Supernatant was harvested after 6 days, and antibodies were purified from a protein A or protein G affinity column (Qiagen). Antigen-binding fragment (Fab) was produced by endoproteinase Lys-C digestion of purified antibody in 25mM Tris-Cl, 1mM EDTA (pH 8) for 6 hours at 37^o^C. The reaction was stopped with 1mM *N*α-*p*-tosyl-L-lysine chloromethyl ketone and 0.4mM leupeptin and the mixture passed over a protein A or protein G column to remove the Fc fragment. Flowthrough fractions containing Fab were concentrated and further purified to remove Lys-C by size-exclusion chromatography (16/60 Superdex-200, GE) in phosphate buffered saline (PBS) or a buffer containing 350mM NaCl, 2.5mM Tris pH7.1, 0.02% NaN_3_.

### RSV Fusion Glycoprotein Construct Design, Expression, and Purification

The RSV F gene constructs from subtype B strains B18537 and B1 were made by synthesis of DNA containing codons 1 to 513 from Uniprot sequence P13843 and O36634, respectively, followed by a T4 fibritin trimerization motif [38], thrombin cleavage site, 6x His-tag, and a StreptagII tag. To stabilize the prefusion state, the F gene was altered to introduce “DS-Cav1” mutations, Ser155Cys, Ser190Phe, Val207Leu, and Ser290Cys. The postfusion RSV B18537 F was made by gene synthesis with codons for fusion peptide residues 137 to 146 deleted from Uniprot sequence P13843 followed by a thrombin cleavage site, 6xHis-tag, and a StreptagII tag.

Soluble RSV F proteins were expressed by transient transfection in Expi293F cells using True-Fect-Max (United BioSystems, MD). The culture supernatant was harvested 5 days post transfection, centrifuged at 10,000*g* to remove cell debris, and then sterile filtered. The supernatant was buffer exchanged and concentrated using tangential flow filtration. RSV F glycoprotein was purified by nickel-(Roche) and Strep-Tactin-affinity (IBA lifesciences) chromatography. Purification tags were removed by thrombin digestion overnight at 4^o^C, and the protein was further purified by size-exclusion chromatography.

### Protein Crystallization and Data Collection

Robotic screening yielded crystals with the RSV strain B18537 F glycoprotein in 1.2M ammonium sulfate, 0.1M sodium acetate pH5.5, 0.1M lithium sulfate. Crystallization conditions were manually optimized in hanging drops by mixing 1 μL of protein complex with 1 μL of the reservoir solution. Crystals were harvested and flash-cooled in liquid nitrogen using 3.2M ammonium sulfate as a cryoprotectant. Data were collected at a wavelength of 1.00 Å at the Southeast Regional Collaborative Access Team (SER-CAT) beamline ID-22 (Advanced Photon Source, Argonne National Laboratory).

### Structure Determination, Model Building, and Refinement

Diffraction data were processed using HKL2000, and crystal unit cell analysis [39] indicated 1 RSV F protomer per asymmetric unit. Molecular replacement searches were carried out with PHASER [40], using as a search model the RSV F subtype A structure (PDB ID:4MMS) [30]. Model building was carried out with Coot [41], and refinement was performed with Refmac5 [42], PHENIX [43], and Buster-TNT [44, 45].

### Structure Analysis and Figure Preparation

The final refined structures were analyzed with MolProbity [46]. Structural comparison and alignments were carried out using LSQKAB [47, 48]. Surface area analyses and contact residue analysis were carried out using PyMOL [49], Pdbsum [50] and PISA [51]. All structural images were created using PyMOL.

### Mice Immunization and Serum Neutralization Assays

Mice were housed and cared for in a facility accredited by the Association for Assessment and Accreditation of Laboratory Animal Care International at the Vaccine Research Center (VRC), National Institute of Allergy and Infectious Diseases, National Institutes of Health in accordance with local, state, federal, and institute policies. Animal experiments were performed in compliance with the Animal Welfare Act requirements for environment enhancement which were adequate to promote the psychological and physical well-being of small animals. The animal care protocol of this study was approved by the VRC Animal Care and Use Committee.

To assess the immunogenicity of DS-Cav1-stabilized RSV F of B18537, we immunized CB6F1/J mice (10 in each group) by intramuscular injection with 10 µg RSV F combined with 50 µg poly I:C adjuvant at weeks 0 and 3. Serum samples were collected at week 5. RSV F B18537 postfusion was used as a control for a side-by-side comparison.

For RSV neutralization assays, sera were serially diluted and mixed with an equal volume of recombinant mKate-RSV expressing F glycoprotein from strain A2 or B18537 and the Katushka fluorescent protein. The mixtures were incubated at 37°C for 1 hour, and 50 μL of each was added to 1.5x10^4^ HEp-2 cells in 30 μL minimal essential medium in each well of 384-well black optical bottom plates. The plates were incubated for 20-22 hours and read with 588 nm excitation and 635 nm emission (SpectraMax Paradigm, Molecular Devices, CA). The EC_50_ for each sample was calculated by non-linear regression using GraphPad Prism (GraphPad Software Inc., CA). *P* values were determined by Student's *t*-test.

### Binding Studies using Biolayer Interferometry

A fortéBio Octet Red384 instrument was used to measure binding kinetics of RSV B18537, B1 and A2 DS-Cav1 F glycoproteins to antibodies AM22, 5C4, D25, MPE8, and motavizumab [34, 35, 52–54]. All assays were performed at 30°C in solid black 96-well plates (Greiner Bio-One), with agitation at 1000 rpm in PBS supplemented with 1% BSA in order to minimize nonspecific interactions [30, 32]. The final volume was 100 μL/well. His-tagged F trimers were captured on Ni-NTA sensors at a capture level between 0.8 and 1 nm with a variability of < 0.1 nm within a row of 8 tips. The biosensor tips were equilibrated for 300 seconds in PBS plus 1% BSA prior to binding measurements in the Fab solutions. Binding reactions proceeded for 300 seconds, followed by 300 seconds dissociation. Dissociation wells were used only once to prevent contamination. Systematic baseline drift was corrected by subtracting the measurements recorded for a sensor loaded with RSV-specific antibodies but incubated with PBS buffer supplemented with 1% BSA. Non-specific binding responses were measured with an HIV-specific antibody 2F5 [55], which does not bind RSV F, and subtracted from RSV antibody-response data. Data analysis and curve fitting were done using Octet software version 7.0. Experimental data were fitted with a binding equation describing a 1:1 interaction. Global analyses of the complete data sets assumed binding was reversible (full dissociation) and used nonlinear least squares fitting, allowing a single set of binding parameters to be obtained simultaneously for all concentrations used in each experiment.

Serum antibody binding analysis was carried out in a similar manner to the kinetic analysis described above. RSV B18537 F DS-Cav1 and postfusion forms were immobilized to NTA bio-sensors through a 6x His-tag. The biosensor probes were then equilibrated for 300 seconds in PBS + 1% BSA buffer. Sera were diluted 50- or 100-fold in PBS + 1% BSA, and binding to the immobilized F proteins was assessed for 300 seconds. To analyze antibody competition for binding F trimers, RSV F-loaded probes were incubated in MPE8 or D25 solutions prior to assays in the diluted sera. Sera depletion was carried out by using 1 μg of DS-Cav1 or postfusion F proteins per 1 μL of animal sera. Parallel correction to subtract non-specific sera binding was carried out by subtracting binding levels of an unloaded probe incubated with the sera.

### Nucleotide Sequence Accession Numbers

The RSV F glycoprotein nucleotide sequences used in this study were downloaded from Gen-bank and the DNA databank of Japan. Accession numbers of utilized sequences are as follows for subtype B strains: JX576729 to JX576762, AF013254 (B1), AY353550 (9320), and for subtype A strains: JQ901447 to JQ901458, JX015479 to JX015499, M74568 (A2), AY911262 (Long), FJ614813 (Line19), NC_001803 (RSS-2), JX482018 to JX482038, JX682715 to JX682823, JX477455 to JX477594.

### Molecular Phylogenetic Analysis

The evolutionary history was inferred by using the Maximum Likelihood method based on the JTT matrix-based model [56]. The tree with the highest log likelihood (-2466.1477) was calculated. Initial tree(s) for the heuristic search were obtained automatically by applying Neighbor-Join and BioNJ algorithms to a matrix of pairwise distances estimated using a JTT model, and then selecting the topology with the superior log likelihood value. The analysis involved 352 RSV F sequences. All positions containing gaps or missing data were eliminated. There were 273 positions in the final dataset. Evolutionary analyses were conducted in MEGA6 [57].

### Sequence Analysis

Sequence variability for RSV subtypes A and B F protein was analyzed using 2 approaches: Shannon entropy and mutual information [58]. A total of 87 (54 A and 33 B) unique RSV F sequences were used in the analyses. Entropy measures the degree of sequence variability for a given residue position, with higher entropy values corresponding to greater variability for that residue, for example, residue 209 has an entropy score of 0.65127 when both sequences from subtypes A and B are assessed, corresponding to a distribution of 64% Lys and 36% Gln, while residue 276 has an entropy score of 0.59977, corresponding to 29% Asn and 71% Ser. Entropy was computed separately for subtypes A and B, as well as for sequences of both subtypes combined.

Mutual information scores take into account the association between mutations at a given RSV F residue position and the prevalence of a residue within a given subtype. Higher mutual information scores are given for a residue where particular mutations are preferentially found in 1 subtype. For example, residue 209 had a mutual information score of 0.56455, which indicates a substantially different amino acid distribution for that residue in RSV F subtype A versus subtype B. Specifically, in RSV F subtype A sequences, residue 209 is a completely conserved Lys, whereas in subtype B, that residue is 94% Gln and only 6% Lys. On the other hand, residue 276 had a mutual information score of 0.171243, indicating the existence of some, though not as pronounced, differences in amino acid distribution between A and B sequences; residue 276 is 46% Asn and 54% Ser in A sequences, whereas it is 100% Ser in B sequences.

## RESULTS

### Expression and Antigenicity of a DS-Cav1 Variant of RSV F Subtype B

**Supplementary Figure 1.**
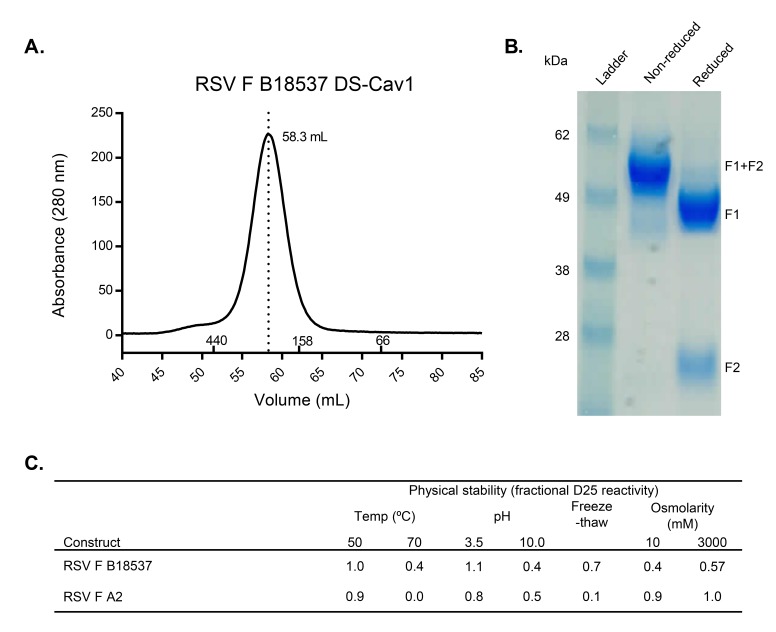
**Purification of RSV strain B18537 DS-Cav1 F glycoprotein.** (A) Size-exclusion chromatograph of RSV B18537 F glycoprotein in PBS buffer on a 120 mL Superdex-200 gel filtration column. Elution volume of protein standards with known molecular weights are indicated along the base of the chromatogram. (B) SDS-PAGE of the peak fraction from size-exclusion chromatography. (C) Physical stability of RSV B18537 F glycoprotein in comparison with those of RSV subtype A F. Physical stability was measured by the fractional value of the D25 reactivity retained after exposure to various physical extremes, compared with the initial D25 reactivity, as determined by Octet biolayer interferometry.

To stabilize the RSV glycoprotein of strain B18537 in its prefusion conformation, we introduced disulfide and cavity-filling mutations (S155C, S190F, V207L, and S290C; termed DS-Cav1 [30]), and appended a trimerization domain from phage T4 (Foldon) [38] to the C terminus of the F ectodomain. The entire coding region was placed into a pAH mammalian expression vector derived from pLEXm [59] and expressed by transient transfection. This DS-Cav1-stabilized RSV F was fully cleaved into the appropriate F2 and F1 polypeptides and eluted from a gel filtration column as a single monodisperse peak at the expected retention volume for a glycosylated trimer (Supplementary Figure 1). Similar results were obtained for an F glycoprotein from strain B1. The typical yield for these DS-Cav1 subtype B RSV F variants was ~1.5 mg of purified trimer per liter of growth media, similar to that of subtype A strain A2 [30]. The purified RSV B18537 F had similar physical stability to RSV A2 F [32] (Supplementary Figure 1C).

We determined antigenic recognition of RSV F using biolayer interferometry (Octet). The F glycoproteins were immobilized on the biosensor tip, and their affinity to a panel of RSV F reactive antibodies was measured (Table 1). Notably, the recognition of both subtypes A and B was similar for most antibodies, except for 3 site Ø-specific antibodies; recognition by antibodies D25 and AM22 was reduced by > 100-fold for a given subtype B strain while antibody 5C4 recognition was fully ablated. Similar results have been reported between another subtype B strain, B9320, and A2 [60]. B18537 has an Arg residue at position 202 located within antigenic site Ø, which is uncommon in subtype B and possibly explains its recognition by AM22; the B1 strain has the more typical Gln residue at position 202. This Arg202 could play a role in AM22 binding similar to Lys201 of subtype A.

**Table 1.** Antigenic characterization of RSV DS-Cav1 F glycoproteins

RSV F Antigen	Antibody Fab/Antigenic site	Binding kinetics		
		*k_on_* (1/Ms)	*k_off_* (1/s)	*K_D_* (M)
B18537		2.99 × 10^4^	2.20 × 10^−2^	7.36 × 10^−7^
B1	D25/site Ø	1.54 × 10^5^	1.66 × 10^−2^	1.07 × 10^−7^
A2		7.76 × 10^4^	1.14 × 10^−5^	1.47 × 10^−10^
B18537		1.59 × 10^4^	< 1.00 × 10^−7^	< 6.29 × 10^−12^
B1	AM22/site Ø	1.77 × 10^4^	2.75 × 10^−4^	1.55 × 10^−8^
A2		1.53 × 10^5^	1.69 × 10^−7^	1.10 × 10^−12^
B18537		NB	NB	NB
B1	5C4/site Ø	NB	NB	NB
A2		2.65 × 10^5^	3.52 × 10^−3^	1.33 × 10^−8^
B18537		8.60 × 10^4^	7.41 × 10^−5^	8.62 × 10^−10^
B1	Motavizumab/site II	1.34 × 10^5^	1.15 × 10^−4^	8.56 × 10^−10^
A2		2.10 × 10^5^	8.59 × 10^−6^	4.09 × 10^−11^
B18537		4.79 × 10^5^	5.89 × 10^−6^	1.23 × 10^−11^
B1	MPE8/site III	2.38 × 10^5^	8.30 × 10^−5^	3.49 × 10^−10^
A2		8.43 × 10^4^	4.04 × 10^−5^	4.79 × 10^−10^

NB = No binding

### Crystal Structure of the Prefusion Form of RSV B18537 F Glycoprotein

Crystallization trials of the DS-Cav1-stabilized strain B18537 F glycoprotein yielded crystals of symmetrical cubes of 50 to 80 μm in size, at pH 5.5 in space group *P*4_1_32 with 1 protomer per asymmetric unit (Table 2). We solved the structure using a starting model from PDB: 4MMS using molecular replacement with Phaser. After iterative cycles of model building and TLS refinement with 6 groups, 454 residues of the RSV F molecule were built in the final refined model. Data collection and refinement statistics are presented in Table 2.

**Table 2.** Crystallographic data and refinement statistics

**PDB accession code**	6Q0S
**Growth condition**	1.2M ammonium sulfate, 0.1M lithium sulfate, 0.1M sodium acetate pH 5.5
**Data collection**	
Space group	*P*4_1_32
Cell constants	
*a, b, c* (Å)	167.9, 167.9, 167.9
α, β, γ (°)	90.0, 90.0, 90.0
Wavelength (Å)	1.00
Resolution (Å)	50.0-1.94 (2.09-2.01, 2.01-1.94)
*R*_sym_	0.10 (0.648, 0.788)
*I* / σ*I*	11.96 (1.94, 1.12)
Completeness (%)	95.9 (96.1, 79.7)
Redundancy	4.0 (3.3, 2.4)
**Refinement**	
Resolution (Å)	50.0-1.94
Unique reflections	57,616
*R*_work_ / *R*_free_ (%)	18.74/21.15
No. atoms	
Protein	3552
Ligand/ion	5
Water	401
*B*-factors (Å^2^)	
Protein	46.2
Ligand/ion	78.3
Water	53.2
R.m.s. deviations	
Bond lengths (Å)	0.017
Bond angles (°)	1.50
Ramachandran	
Favored regions (%)	95.3
Allowed regions (%)	4.5
Disallowed regions (%)	0.2

Values in parentheses are for the highest-resolution shells. R_sym_ = Σ|I − <I>|/Σ<I>, where I is the observed intensity, and <I> is the average intensity of multiple observations of symmetry-related reflections. R = Σhkl||Fobs| − |Fcalc||/Σhkl|Fobs|. R_free_ is calculated from 5% of the reflections excluded from refinement.

**Figure 1.**
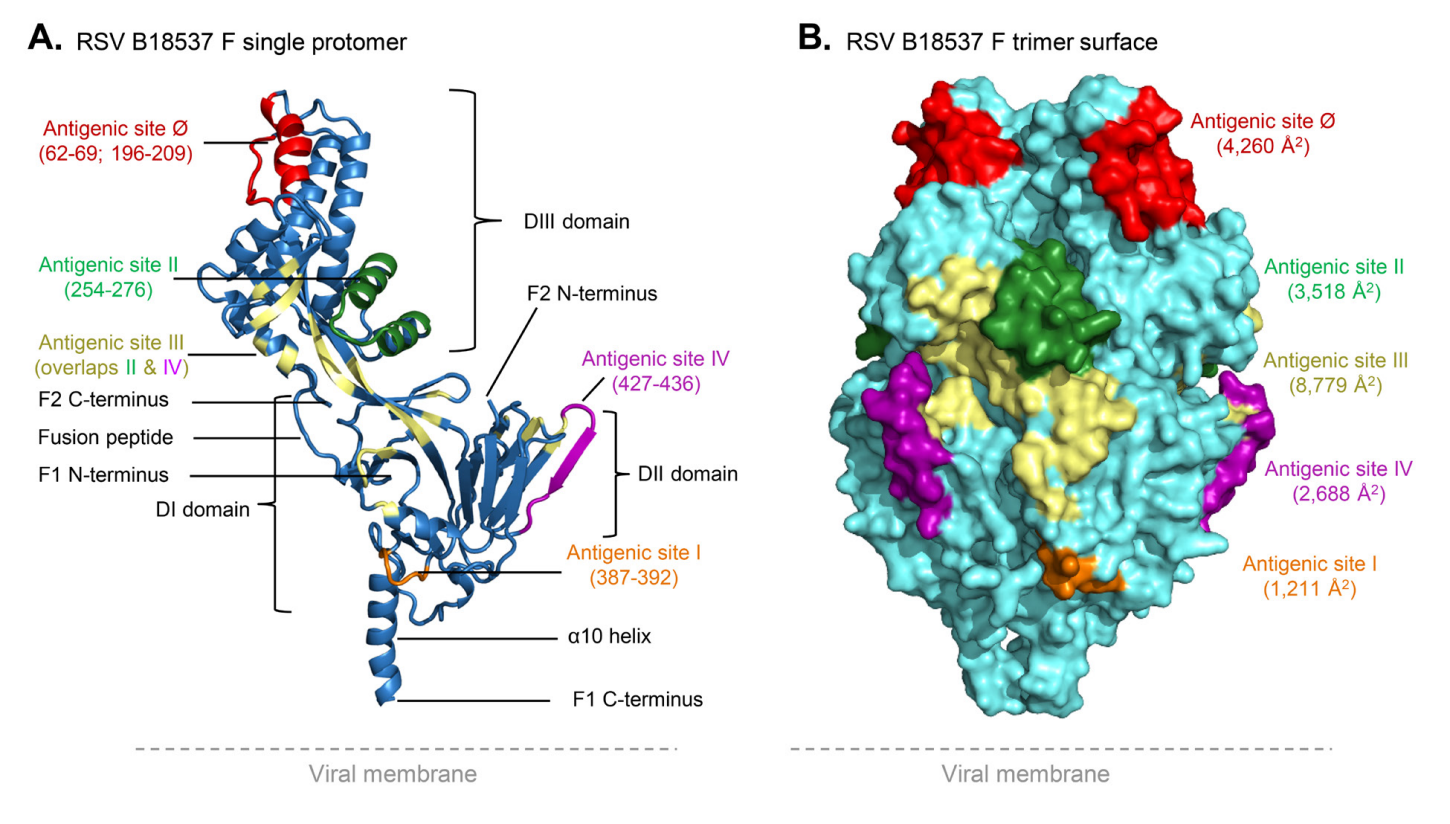
**Crystal structure of the fusion glycoprotein of RSV B18537.** (A) Ribbon representation of a single protomer of the RSV B18537 F glycoprotein. RSV F domains, antigenic sites, fusion peptide, and subunit termini are labeled. Antigenic site III is discontinuous and overlaps with sites II and IV. The residues of site III overlapping with site II or IV are colored only as site II or IV, respectively. Another known antigenic site, site V [65], located between sites Ø and II and overlapped with sites Ø, II, and III, is not shown for clarity. (B) Surface representation of the RSV B18537 F trimer with the antigenic sites colored as in (A) and the antigenic surface area (Å^2^/protomer) indicated. The total trimeric accessible surface area (residues 18 to 509) is 52,225 Å^2^, calculated using a surface accessibility radius of 1.4 Å.

The F glycoprotein formed a homotrimer, each protomer comprising 2 different subunits, F2 and F1 (Figure 1). Residues 26 to 106 of the F2 subunit as well as residues 137 to 509 of the F1 subunit were well defined by the experimental electron density. Each protomer comprised 4 major structural domains (Figure 1A), the membrane proximal α10 helix, the DI domain including the F2 N-terminus, the DII domain (residues 401-463), and the membrane distal DIII domain (residues 49-308) including the F2 C-terminus. Each protomer had major interactions (2250 Å^2^ involving ~ 75 residues) with neighboring protomers resulting in an interweaved structure, where the DIII domain was located directly above the DII domain of an adjacent protomer. The fusion peptide of each protomer was located in a central cavity, interacting with adjacent protomers. We determined the surface areas of antigenic sites on the prefusion trimer using the previously defined antigenic site residues [35, 61–65] (Figure 1B). Antigenic site III had the largest area at 8,779 Å^2^, and it overlapped with sites II and IV. Antigenic site V [66], also defined as site VIII [67], overlapped with sites II, III, and IV.

**Figure 2.**
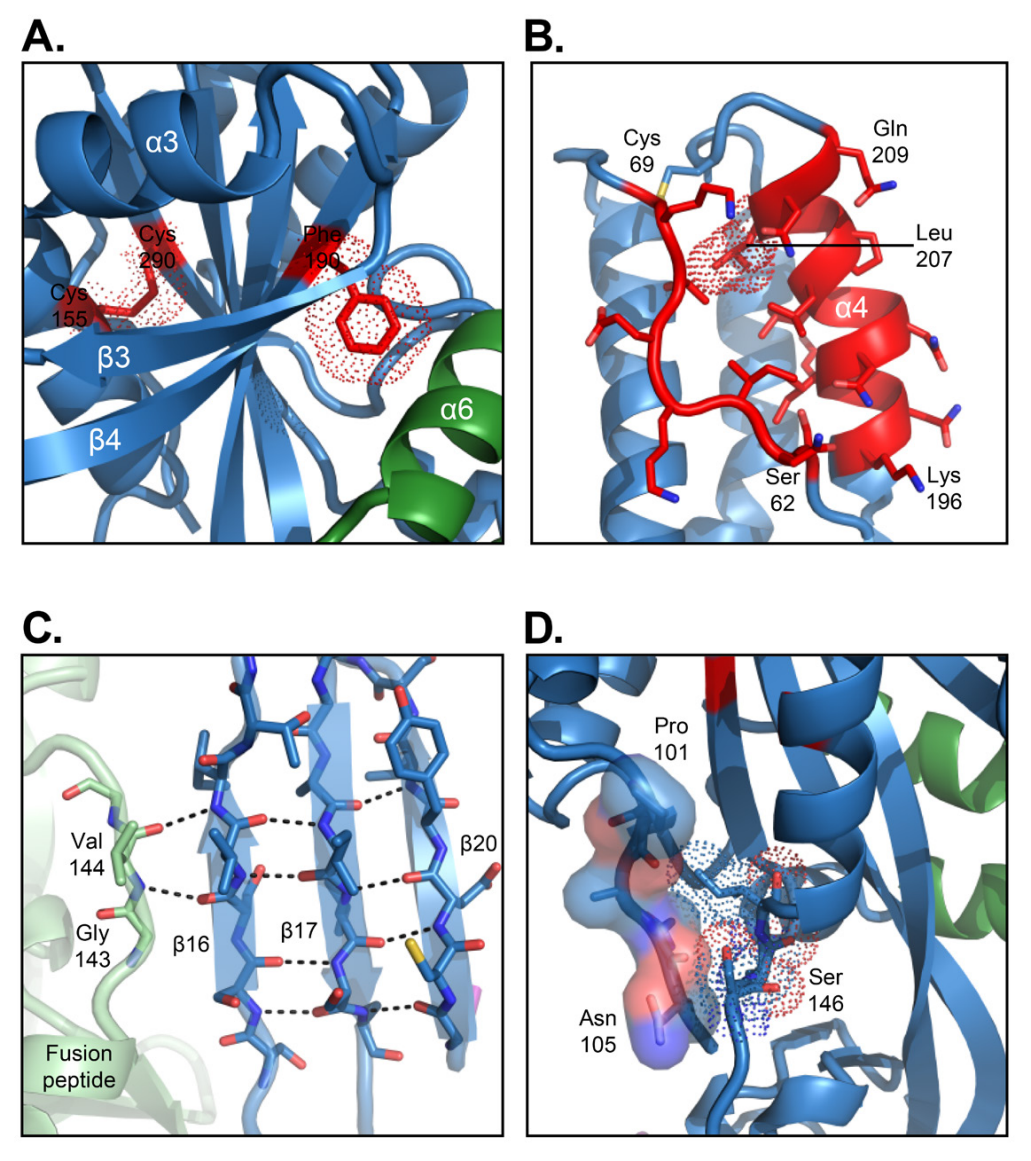
**Atomic-level details of the RSV B18537 F glycoprotein structure**. (A) Prefusion stabilizing DS-Cav1 mutations (S155C, S190F, S290C) are shown with van der Waals surface (red dots) with the adjacent secondary structure including the domain III β-propeller fold shown in ribbon format. (B) Antigenic site Ø located at the apex of each protomer is highlighted in red with side chains in stick representation. The V207L mutation, which is part of the stabilizing mutations, is highlighted in dot representation. (C) Inter-protomer hydrogen bonds between the fusion peptide of one protomer and β strands 16, 17, and 20 from an adjacent protomer are indicated by dotted lines. (D) Interaction between the F2 C-terminus and the fusion peptide within a single protomer. The F2 C-terminus is shown in surface representation and the fusion peptide shown with dotted van der Waals surface.

**Supplementary Figure 2.**
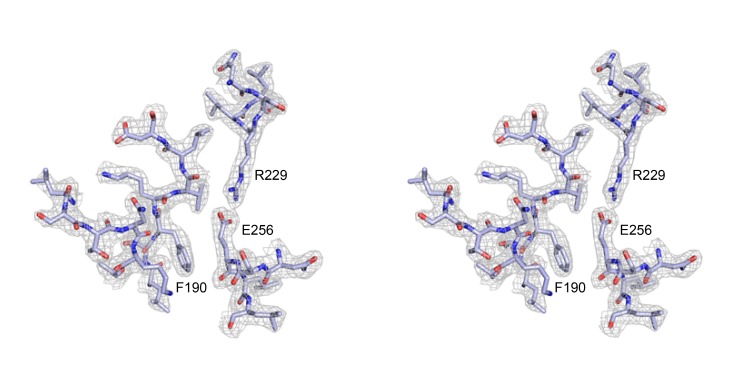
**Electron density of RSV strain B18537 DS-Cav1 F glycoprotein around the Cav1 residue Phe190 shown in stereo image.** The 2F_o_-F_c_ electron density map is contoured at 1 α and shown in gray mesh format. Amino acid residues are shown in stick representation.

Most of the DS-Cav1 mutations were clearly visible in electron density maps, as exemplified by the S190F mutation shown in Supplementary Figure 2. The S155C and S290C mutations formed a prefusion-stabilizing disulfide bond (Figure 2A). The Cav1 mutations S190F (Figure 2A) and V207L (Figure 2B) stabilized intra-protomer interactions. The interweaved nature of the protomers was apparent at the local level where fusion peptide residues 143 and 144 main-chain hydrogen-bonded with domain II strands β16 of an adjacent protomer (Figure 2C), while Ser146 to Ile148, located just after the fusion peptide, interacted with the F2 C-terminal region of the same protomer (Figure 2D).

**Figure 3.**
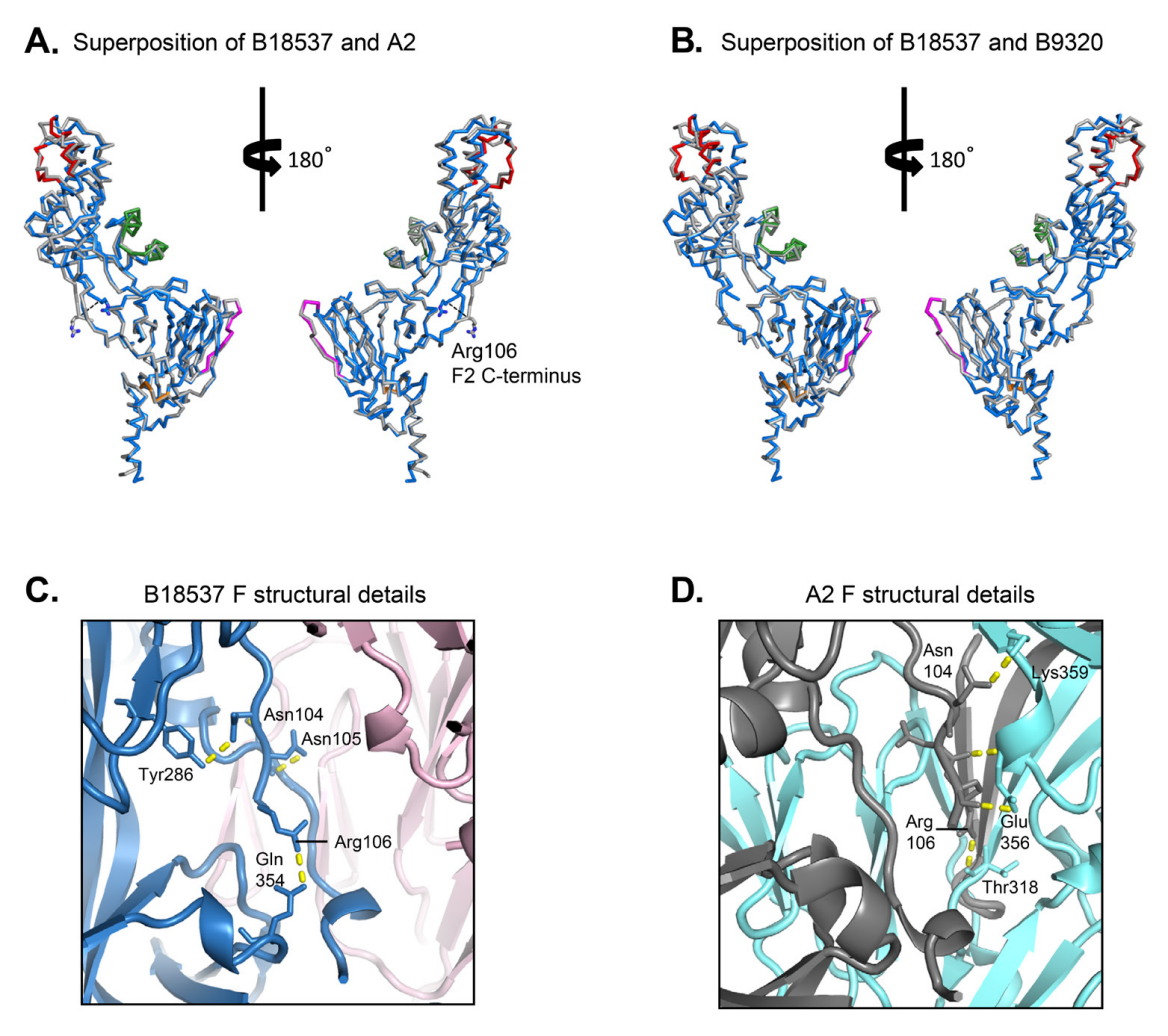
**Structural comparison of RSV strain B18537 DS-Cav1 F glycoprotein with A2 DS-Cav1 F (PDB ID: 4MMU) and with B9320 DS-Cav1 F (PDB ID: 5UDE).** (A) Structural superposition of a protomer of B18537 F (colored as in Figure 1) with that of A2 (gray) shown in ribbon representation. The 2 structures have a RMSD of 1.18 Å over 454 Cα atoms. There is a shift of 8.5 Å between the F2 C-termini of B18537 and A2 F glycoproteins. The F2 C-terminus Arg106 residues of the 2 strains are shown in stick representation with a dashed line indicating the distance between them. (B) Structural superposition of a protomer of B18537 F with that of B9320 (gray). The 2 structures have a RMSD of 1.06 Å over 444 Cα atoms. (C) Close-up view of the F2 C-terminus of the B18537 F structure and its interactions with the fusion peptide and other parts of the same protomer. Shown in pink is an adjacent protomer. (D) Close-up view of the F2 C-terminus of the A2 F structure and its interactions with an adjacent protomer (cyan). In (C) and (D), yellow dashes indicate hydrogen-bonds.

In general the structures of the RSV subtypes A and B F glycoproteins were very similar. The root-mean-square deviation (RMSD) between the structures of B18537 (determined here) and A2 (PDB ID: 4MMU) was 1.18 Å over the Cα atoms of 454 residues present in both structures. Despite 28 residues being different in the mature sequence between the 2 proteins, their overall secondary structures were well conserved (Figure 3). Most of the differences were at the apex and F2 C-terminal region. In both structures, residues 101 to 106 of the F2 C-terminus were located adjacent to the fusion peptide of the same protomer (Figure 3C, D); in the A2 structure, however, this region also interacts with a short helical region from residues 354-358 in an adjacent protomer, resulting in a shift of 8.5 Å of Arg106 between the 2 structures (Figure 3A, D). Both the F2 C-terminus and F1 N-terminus were in the central cavity of the RSV F glycoproteins.

**Figure 4.**
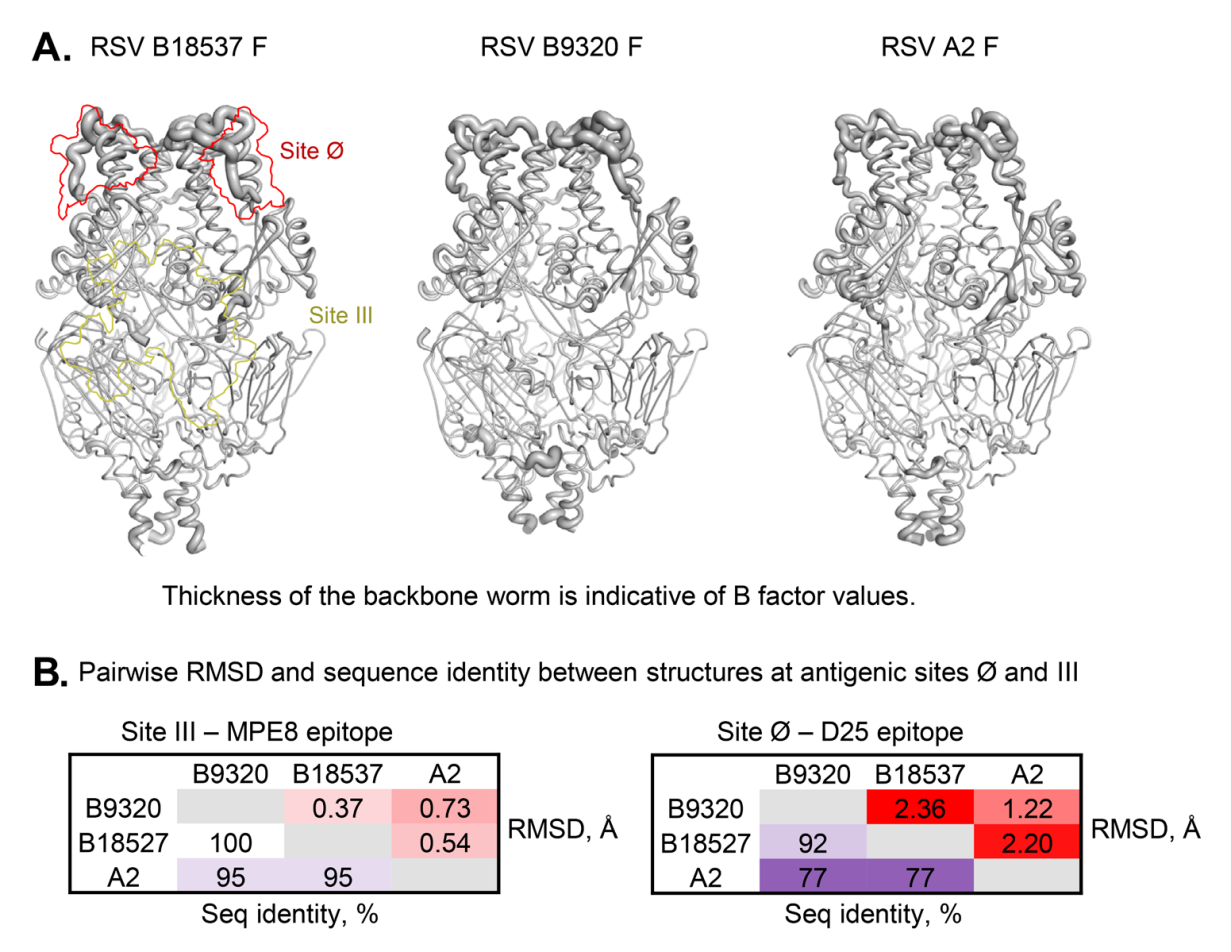
**DS-Cav1 stabilized RSV F glycoproteins have higher flexibility at the apical region**. (A) Worm diagrams of the RSV F trimers with thickness proportional to B factor values. The figures were prepared with PyMOL from crystal structures of B18537, B9320 (5UDE), and A2 (4MMU). The positions of antigenic sites Ø and III on the front face are marked with red and pale-yellow contours on the diagram of the B18537 structure. (B) Pairwise RMSD between the 3 structures at 2 antigenic sites with the corresponding sequence identity of the epitope residues. RMSD was calculated based on Cα atoms.

Comparison between the 2 structures of RSV F subtype B, B18537 and B9320 [37], showed that they had an RMSD of 1.06 Å over 444 Cα atoms present in both structures, even though the 2 proteins are > 99% identical in sequence. Similar to that described above between subtypes A and B, most of the differences were at the apical region and termini where the B factors were high (Figure 4), indicating these regions to be structurally flexible. Some of the observed variation was likely to be due in part to the low resolution of the B9320 structure, whose F2 C-terminal region is partially disordered, missing residues 100-106, which are present in the B18537 F structure. One notable difference was at the F1 N-terminal region, whose sequence is conserved between the 2 subtype B strains (detailed below). This region is involved in the interface between protomers.

### Analysis of F Glycoprotein Subtype Sequences

**Figure 5.**
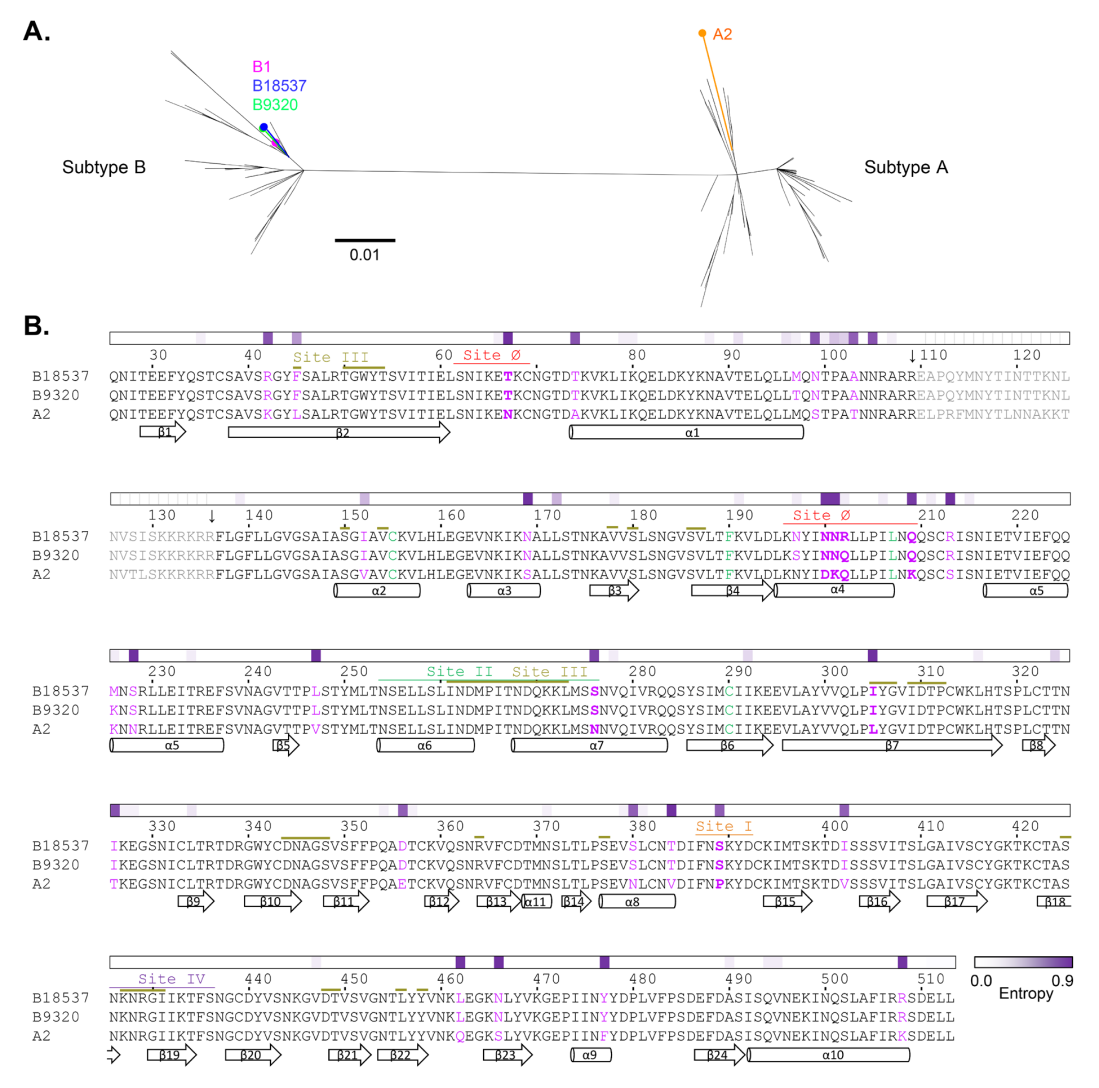
**RSV F phylogeny, subtype sequence alignment, secondary structure definition, and sequence entropy.** (A) Molecular phylogenetic analysis of RSV F sequences by Maximum Likelihood. The tree was drawn to scale, with branch lengths measured in the number of substitutions per site. Strains B1, B18537, B9320, and A2, are labeled. (B) Sequence alignment of RSV F proteins from B18537, B9320 and A2. Antigenic sites Ø, I, II, III, and IV are marked above the sequences with color-coded lines. Site III (colored pale yellow) is discontinuous and has substantial overlaps with sites II and IV. Residues that differ between the strains are highlighted in purple and those also found in antigenic sites are highlighted in bold. The DSCav1 mutations (S155C, S190F, V207L, S290C) are highlighted in green. Secondary structural elements of RSV F as defined from the B18537 structure are shown below the sequence alignment. Sequence entropy calculated from 190 RSV F sequences is plotted above the sequence with a color gradient according to the color key shown at the lower right. Residues between 2 arrows (residues 110-136) are removed by protease digestion and therefore not present in the mature proteins.

Analysis of 352 RSV F sequences (partial or complete; 109 complete B sequences and 164 complete A sequences) showed that subtype A and subtype B sequences were separated into 2 distinct phylogenetic branches (Figure 5A). From this phylogenetic tree, it was clear that RSV subtypes could be categorized based on F sequences, in addition to the conventional G sequence-based subtyping [68]. Subtypes A and B F glycoproteins differed by ~5%-10% from each other in amino acid sequence, and B18537 F differed from A2 F by 38 residues, including 10 in the 27-residue peptide between furin-cleavage sites (Figure 5B). Within each subtype there was significantly less sequence variation, while there was slightly more diversity among A strains than among B strains. The high-resolution structure of B18537 F allowed for a clear definition of the secondary structure of the RSV F glycoproteins, thereby updating the nomenclature of the loop, helices, and β-strands (Figure 5B). There are several well-characterized antigenic sites on the F glycoprotein [69], site I (residues 387-392) targeted by 2F and 131-2a, site II (residues 254-276) targeted by palivizumab, site III (residues 45, 50-54, 150, 154, 178, 180, 186-187, 261-273, 305-307, 309-312, 344-347, 364, and 377 from one protomer and residues 425, 427-431, 448-449, 456 and 458 from an adjacent protomer) targeted by MPE8, site IV (residues 427-436) targeted by 101F, and site Ø (residues 62-69 and 196-209) targeted by D25, AM22, and 5C4. Sequence entropy analysis indicated differences among the strains to be evenly spaced throughout the sequence. There was only 1 residue with significant entropy in sites I (residue 389), II (residue 276), and III (residue 305), while none was observed in site IV. By contrast, there were 5 residues in the prefusion-specific site Ø with significant entropy (Figure 5B). This scarcity of sequence variation in most antigenic sites except site Ø has been observed in an analysis of RSV clinical isolates [70].

### Surface Antigenic Differences Between RSV B18537, B9320, and A2 F Glycoproteins

**Figure 6.**
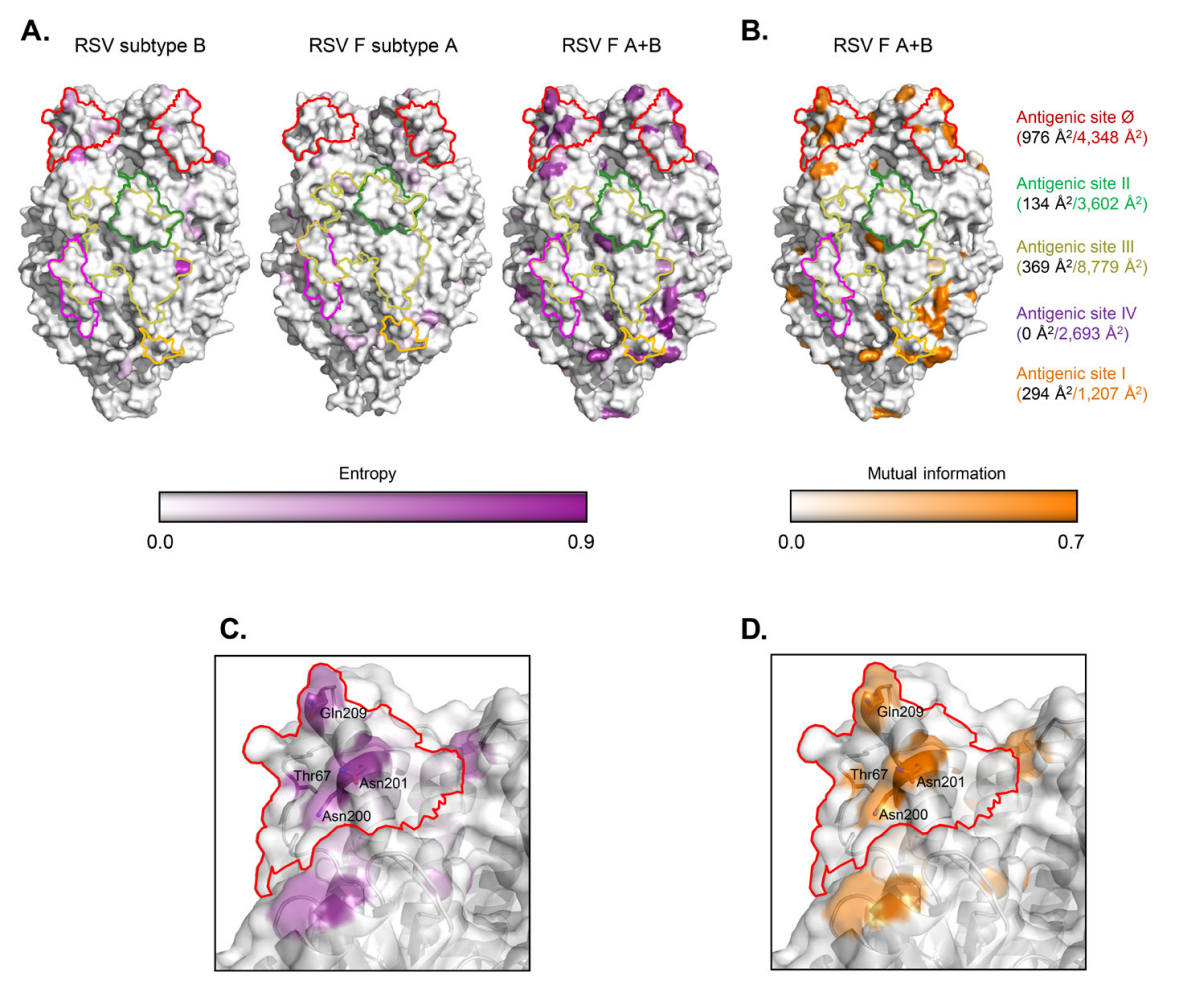
**Surface antigenic differences between subtypes A and B**. Surface antigenicity does not differ significantly within a subtype, but significant differences are observed between subtypes, with antigenic site Ø having approximately 23% surface variation. (A) Sequence entropy mapped on protein surface with a purple color scale as shown. (Left) Residue differences found within subtype B are mapped on the B18537 F structure; (Center) differences within subtype A are mapped on the A2 F structure; (Right) differences between subtypes A and B are mapped on the B18537 F structure. The antigenic sites are outlined with borders colored as in Figure 1. (B) Mutual information comparison of subtype B and subtype A sequences mapped on the B18537 F structure. Total antigenic site surface areas (Å^2^) as shown in Figure 1 are indicated to the right and colored the same as the antigenic site outline. Differences between subtypes are indicated in black fonts for each antigenic site. (C) Zoom-in view of subtype differences at antigenic site Ø as defined by sequence entropy. (D) Antigenic site Ø differences between subtypes A and B as defined by a mutual information metric. In (C) and (D), transparent trimer surface is shown overlaid on the cartoon representation with side chains shown in sticks for residues with significant difference.

Shannon entropy and mutual information analysis of subtypes A and B F sequences indicated that subtype-specific residues accounted for an approximate total of 5700 Å^2^ of solvent accessible area, which is approximately 11% of the F trimer surface (Figure 6). These surface changes were spread throughout the protein, but there were 2 regions where differences clustered. The first region (residues 42, 326, 380, 384, 466, and 477) was in the DII domain, which is similar in structure in both prefusion and postfusion conformations. However, to date, there have been no neutralizing antibodies mapped to this region. The second region included antigenic site Ø, which has large structural changes between the prefusion and postfusion conformations, and, as noted above, is a focus of potent RSV-neutralizing human antibodies.

Antigenic site I antibodies 2F, 44F, and 45F are subtype specific, with neutralization dependent on the residue 389 (Figure 5B). This residue is Pro in subtype A strains sensitive to 2F, 44F, and 45F, while subtype A strains with Ser/Leu/His at 389 are resistant; Ser389 is conserved in all subtype B strains, preventing their neutralization by 2F, 44F, or 45F [71, 72]. In antigenic site II, Ser276 is highly common in subtype B including B18537 [14], whereas Asn is found at 276 in A2. Residue 276 is at the edge of the site II epitope with its side chain facing away from the bound antibody and making minimal contacts [61]; this residue is not involved in recognition by site II-specific antibodies. As shown in Table 1, the antigenic recognition of B18537 F by motavizumab (an optimized version of palivizumab) was comparable to that of A2 F. There have been reports of subtype B resistance to palivizumab, but in addition to the N276S change, there is a requirement for a K272E mutation [73] or N268I and N276Y mutations [74]. Lastly, the sequence of antigenic site IV on the DII domain was fully conserved in all strains analyzed.

The prefusion-specific antigenic site Ø, which is located at the apex of the F glycoprotein, differed between B18537 and A2 in 5 of the 22 residues of this antigenic site (Figures 5B and 6), and structural alignment analysis indicated an RMSD of 2.2 Å in this region (Figure 4B). This subtype difference at site Ø was consistent with results from antigenic analysis; 2 of the 3 site Ø-specific antibodies revealed a significant difference in binding affinity (Table 1). D25 had an affinity ~100-fold lower against B18537 F than against A2 F; 5C4 was unable to bind B18537 F, whereas it bound A2 F with a nanomolar affinity. Structural variation at site Ø is partly due to structural flexibility at the apex of RSV F prefusion structures, as revealed by the high B factors at this region for all 3 structures (Figure 4A). The RMSD of site Ø residues is greater between B18537 and B9320 than between B18537 and A2, despite the sequence identity being greater between B18537 and B9320 (Figure 4B). Thus, antigenic differences were minor between strains B18537 and A2 F glycoproteins, except at antigenic site Ø, where residues of higher entropy clustered and substantial differences in affinity were observed for several of the antibodies that target this site.

### Prefusion-Stabilized RSV B18537 DS-Cav1 F Elicits High Neutralization Titers Against Both Subtypes A and B Viruses

**Figure 7.**
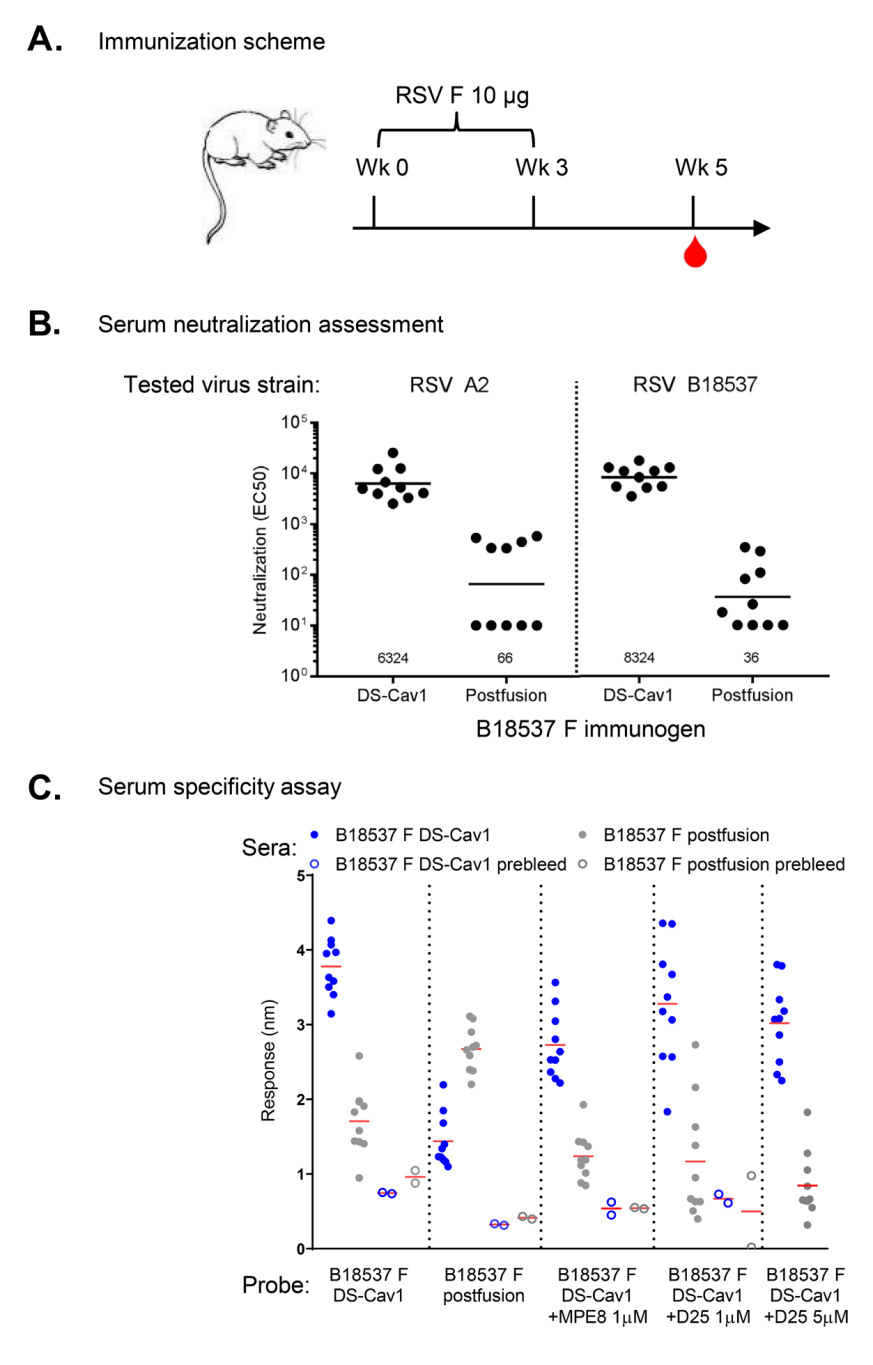
**Prefusion-stabilized RSV B18537 DS-Cav1 F elicits high neutralization titers against both subtypes A and B viruses.** (A) Mice were immunized with either DS-Cav1 or postfusion B18537 F glyco-protein at weeks 0 and 3. Serum samples were taken at week 5. (B) Mouse sera were assayed for neutralization against subtype A and B strains. Each dot represents the serum titer from a single mouse. Average titer of each group is marked with a line and also labeled on the horizontal axis. (C) Sera from mice immunized with RSV B18537 F prefusion DS-Cav1 or postfusion immunogen were assessed for binding to the immobilized RSV B18537 F DS-Cav1, postfusion, DS-Cav1 bound by MPE8, or DS-Cav1 bound by D25. Average response of each group is marked with a red line. Statistical analyses in (B) and (C) were calculated by Mann-Whitney 2-tailed *t* test, ****: *P* < 0.001.

To evaluate the potential of RSV subtype B F glycoprotein as a vaccine candidate, we immunized mice with prefusion-stabilized B18537 DS-Cav1 F and analyzed the serum immunogenicity (Figure 7). The DS-Cav1-immunized sera had more than 100-fold higher titers than those immunized with B18537 postfusion F, when assessed against either the homologous B18537 virus or against the heterologous A2 virus. In contrast to the immunization results from subtype A DS-Cav1 [30], the prefusion F subtype B-immunized sera neutralized both subtypes A and B at comparable levels (Figure 7B). Assessment of serum antigenicity for binding to prefusion or postfusion probes indicated serum responses to be specific to the conformation of both immunogen and probe (Figure 7C). We further delineated the prefusion-specific responses, by using prefusion-specific antibodies MPE8 (which binds to an equatorial site) and D25 (which binds to an apical site). We observed MPE8 at 1µM to reduce substantially the recognition by DS-Cav1-immunized sera. D25, which binds B18537 F DS-Cav1 at a lower affinity, was able to reduce the recognition by DS-Cav1-immunized sera to a similar level at 5µM, a concentration substantially higher than its *KD* (Table 1). These results suggested that the immunization-elicited responses to B18537 F DSCav1 were likely to be directed equally to both apical and equatorial sites.

## DISCUSSION

Production of a safe and effective vaccine against RSV is a major worldwide health objective, and elicitation of neutralizing antibodies that target the F glycoprotein may be the most likely avenue for achieving this goal (reviewed in [9]). The structure of the prefusion form of the RSV B18537 F glycoprotein provides insights to the antigenic differences and immune responses observed in patients. Of the antigenic sites defined to date, site Ø is the most heavily altered between the 2 major subtypes. These alterations do not change the overall secondary structure of the antigenic site, but they do affect antibody recognition. Given that this site is targeted by potent RSV-neutralizing antibodies that appear to make up the majority of the RSV-neutralizing response in humans, site Ø differences may provide an explanation for the lack of subtype cross-protection. Further understanding of site Ø-specific antibodies and their mechanism of recognizing F glycoproteins from both subtypes may be helpful to further efforts at vaccine design, as the isolation and characterization of antibodies from immunized animals or from infected patients should allow us to understand why infection with one subtype does not fully protect against infection from the other subtype.

The RSV-neutralizing response elicited by B18537 prefusion-stabilized F in mice appeared not to be focused entirely on site Ø. Rather, equal responses against both equatorial and apical sites were observed, strongly competed by both MPE8 and D25. This targeting, together with the high conservation of sequence and structure in the equatorial regions, provide an explanation for the similar neutralizing responses elicited by B18537 DS-Cav1 F against both RSV subtypes. It remains to be seen if similar equatorial responses will be elicited by B18537 DS-Cav1 F in humans.

**Figure 8.**
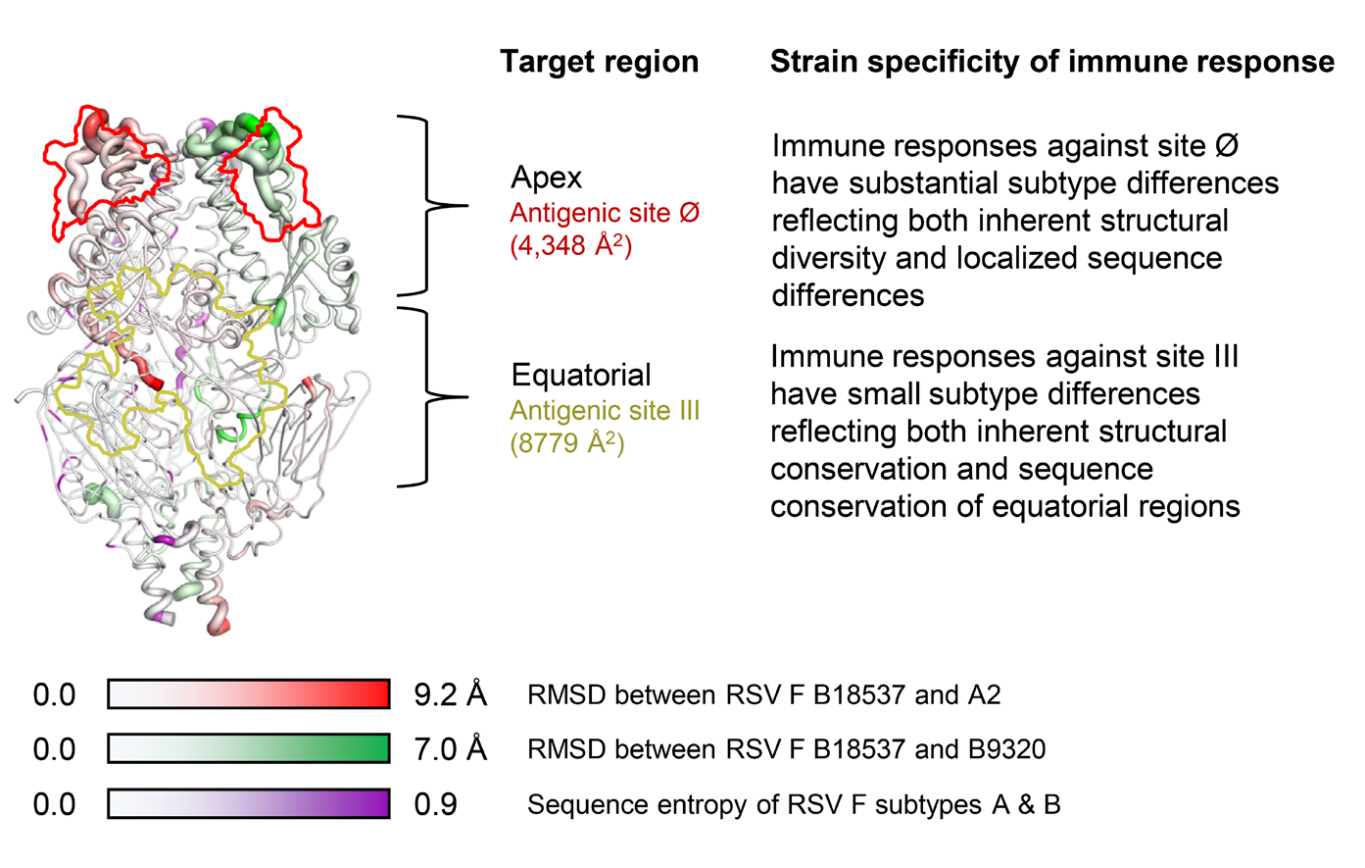
**Schematic of RSV F highlighting structural features and subtype differences in immunogenicity.** A composite RSV F trimer comprising a subunit each from the structures of B18537, B9320 (PDB 5UDE), and A2 (PDB 4MMU) shows a shape similar to the individual structures. Each subunit is shown as worm representation with thickness indicating B factor values. On the subunit of B18537 is mapped the RMSD between the structures of B18537 and A2 F in a gradient of red; on that of B9320, the RMSD between the structures of B18537 and B9320 F in a gradient of green; and on that of A2 is mapped the sequence entropy of RSV F subtypes A and B sequences in a gradient of purple. The positions of sites Ø and III on the front face are marked with contours in red and pale-yellow, respectively.

The availability of prefusion-stabilized RSV F glycoproteins from subtypes A and B should enable the assessment of dual subtype immunization mixtures, the sequential immunizations of subtypes, as well as other immunization regimens aimed at generating improved neutralizing titers against both subtypes. In terms of optimizing the vaccine-generated protective response, the high-resolution RSV B18537 structure should allow vaccine designers to incorporate sub-type differences into structure-based efforts. The RSV F prefusion structures are well conserved between subtypes, with the observed structural differences resulting from sequence variation as well as the high flexibility at the apical regions (Figure 8). It may be possible to generate an RSV F glycoprotein with a chimeric antigenic site Ø that can elicit improved neutralization breadth against both subtypes A and B strains. A self-assembling nanoparticle displaying DS-Cav1-stabilized RSV F induces 10-fold more potent serum neutralizing responses than the trimeric DS-Cav1 RSV F [75]; that use of a dual-component nanoparticle, such as insect ferritin [76], to present prefusion-stabilized RSV F of both subtypes A and B on a single nanoparticle, could increase the potency of responses against both subtypes.
